# Measurement of mandibular incisive canal diameter using cone beam computed tomography

**DOI:** 10.6026/973206300200394

**Published:** 2024-04-30

**Authors:** Prachi Anand, Dhaval N Mehta, Dewanshu Kumar, Richa Thakur, Rohit Malviya, Rohit Kumar

**Affiliations:** 1 Department of Oral Medicine and Radiology, Narshinhbhai Patel Dental College and Hospital, Sankalchand Patel University, Visnagar, Gujarat, India; 2 Department of Prosthodontics Crown and Bridge, Maharana Pratap Dental College and Hospital, Kanpur, Uttar Pradesh, India; 3 Department of Oral Medicine and Radiology, Dental College of Azamgarh, Purvanchal University, Azamgarh, Uttar Pradesh, India; 4 Department of Prosthodontics Crown and Bridge Career Post Graduate Institute of Dental Sciences, Dr.Ram Manohar Lohia Avadh University, Ayodhya, Uttar Pradesh, India; 5 Department of Oral medicine and Radiology, Bhabha college of dental sciences, Bhabha University, Bhopal, Madhya Pradesh, India

**Keywords:** Cone beam computed tomography, Mandible, diameter

## Abstract

Cone beam computed tomography was used for measuring the diameter of a Mandibular incisive canal. The dental practice can now visualize small bone structures with low dose and high spatial resolution due to the introduction of CBCT (Cone Beam Computed Tomography). Therefore, it is of interest to measure the diameter of the MIC (Mandibular Incisive Canal) using the CBCT. Fifty CBCT scans obtained for implant placement preoperative planning were included in the study material. Observers carefully examined the CBCT data to determine the MIC's presence & position. The inner diameter of the MIC was measured by taking the longest distance between the inner cortical borders of the canal which was further analyzed using software CS3D software. The collected data were further subjected to statistical analysis. The average mean of the population was 1.7130±.5178. The average diameter of MIC in males was 1.735±.5406 and in females was 1.6800±.4934. Complications from implant surgery in the anterior mandible may be prevented by utilizing CBCT scans to analyze the smallest diameter of the MIC.

## Background:

Surgery is usually regarded as safe in the anterior mandible. However, damage to the lingual vascular canals, lingual concavity, and mandibular incisive canal (MIC) during surgical procedures may result in haemorrhagic complications and neurosensory disturbances [1, 2 & 3]. Medially extending from both MF ("Mental Foramens"), between the lingual & vestibular cortical plates, is the bilateral canal known as the MIC, which carries a neurovascular bundle. Many benefits have been added to traditional two-dimensional methods by the most recent development in CBCT imaging. The ones that are most frequently mentioned are the removal of neighbouring structure superimposition, the lack of picture magnification, the quick scanning time, and the lower radiation exposure. The opportunity to see small details of the fine canal through the bone structures with high spatial resolution as well as the low dose exposure is provided by the introduction of CBCT into dental practices [4, 5, 6 & 7]. Still, not many studies are available for evaluation of MIC with CBCT. In addition, not much data on age range differences and gender wise difference in MIC diameter has been presented till date.

## Materials and Methods:

50 CBCT images were retrospectively collected from the CBCT centre in Kanpur. All these images were made with "a CS9300 3Dunit, with field of view of 8cmx8cm, voxel size - 800µm, X-ray pulse time of 30ms, kVp -85 kV (max), mA -7 Ma, exposure time of" 10.8s. Informed consent was given by each patient before the examination. Subjects involving mandible scans were included in the study. Syndromic patients & congenital deformity cases, History of trauma, pathology, and surgery involving mandible and Distorted or blurred CBCT images were not considered. The data were reconstructed in panoramic view and MICs were identified and measured with CS 3 D SOFTWARE for visualization of diameter. Axial, panoramic, as well as reformatted cross-sectional images were thoroughly investigated.

## Data and statistical analysis:

0.05 Or below P-values were considered statistically significant. All data were collected and statistically examined using SPSS 16.0. A t-test was performed to evaluate major differences between genders.

## Results:

A total of 50 scans were taken of which 23(46%) were in the age group of 20-40, 26(52%) were in the age group of 40 -60 and 1(2%) lay above 60 years (Table 1). The mean diameter of 20 to 40 years was 1.6196±0.61, in 40-60 years was 1.7808±.423 and age 60 years and above was 2.1 (Table 1). The average mean diameter of the population was 1.7130±.5178 (Table 1). The diameter ranged from 0 to 3mm. The comparison among the age groups "was not statistically significant (p>0.05) (Table 1). The average diameter of the mandibular incisive canal in males was 1.735±.5406 and in females was 1.6800±.4934. The comparison of males and females was not statistically significant (p >0.05) (Table 2).

## Discussion:

A total of 50 scans were used for analysing the diameter of MIC in CBCT. Similar studies were conducted by Dimitar *et al.* (2013) in which 140 CBCT scans was used. In our study diameter was categorized for various age ranges which was not done in any studies. The average mean diameter of the population in our study was 1.7130±.5178 mm. The measurement was nearly similar to the findings of Dimitar *et al.* (2013) in which the diameter was 1.44 ±0.39mm. In other studies, "the mean internal diameter of the incisive canal found was 1.3mm in the Bavitz *et al.* study [8], and 1.8 mm in the Jacobs *et al.* study [9]. In our study, the diameter ranged from 0 to 3mm. Obradovic *et al.* observed that the MIC varied from 0.48mm to 2.9mm on cadaver mandibles; Pires *et al.* found diameters from 0.4mm to 4.6mm on CBCT scans; diameters of 1.0mm to 6.6mm on CBCT" investigations were recorded by Uchida *et al.* [7]. Various factors, including differences in study design, technical issues like pixel size and device variability, as well as individual peculiarity, can account for discrepancies in CBCT results. The variations in diameter and distance to the cortical bone highlight the importance of using CBCT for preoperative evaluation of the MIC in all cases.

## Conclusion:

Mandibular incisive canal is an almost permanent finding on CBCT scans. Mean diameter is non-significantly increasing as age increases in this study. No significant difference was found in diameter of mandibular incisive canal between male and female in this study. Multicentre studies with larger sample size required to establish difference between various age groups and both gender. Preoperative evaluation of mandibular incisive canal can be helpful in accurate treatment planning and prevention of complications.

## Figures and Tables

**Table 1:** The table shows the mean of different age group with the comparison among them

		**N (%)**	**Mean**	**Stand Deviation**	**p value**
Average diameter of MIC	20-40	23(46%)	1.6196	0.61028	0.425
	40-60	26(52%)	1.7808	0.4231	
	60 AND ABOVE	1(2%)	2.1	.	
	Total	50	1.713	0.51783	

**Table 2:** The table shows the range of the mean diameter among genders with comparison

**Group Statistics**					
	**GENDER**	**N**	**Mean**	**Std. Deviation**	**P VALUE**
Average diameter of MIC	MALE	30	1.735	0.54062	0.451
	FEMALE	20	1.68	0.49348	

## References

[1] Dimitar Y (2014). Biotechnology & Biotechnological Equipment..

[2] Abarca MD (2006). Clin. Oral Investig..

[3] Bartling R (1999). J. oral Maxil. Surg..

[4] Makris NPF (2010). Clin. Oral Implan. Res..

[5] Pires CA (2012). Clin. Implant Dent. Res..

[6] Jaju PP (2014). Clin Cosmet Investig Dent..

[7] Uchida Y (2009). J. oral Maxil. Surg..

[8] Bavitz JB (1993). Int. J. oral Max. Implants..

[9] Jacobs R (2002). Dentomaxillofac. Rad..

